# Ectopic third molar in the mandibular condyle: A review of the literature

**DOI:** 10.4317/medoral.17864

**Published:** 2012-08-28

**Authors:** Fernando Iglesias-Martin, Pedro Infante-Cossio, Eusebio Torres-Carranza, Victoria-Eugenia Prats-Golczer, Alberto Garcia-Perla-Garcia

**Affiliations:** 1 Resident, Department of Oral and Maxillofacial Surgery, Virgen del Rocio University Hospital, Seville, Spain; 2 Professor, Department of Oral and Maxillofacial Surgery, Virgen del Rocio University Hospital, Seville, Spain; 3 Staff Surgeon, Department of Oral and Maxillofacial Surgery, Virgen del Rocio University Hospital, Seville, Spain; 4 Associate Professor, Department of Oral and Maxillofacial Surgery, Virgen del Rocio University Hospital, Seville, Spain

## Abstract

Objectives: To evaluate the etiopathogenesis, clinical features, therapeutic options, and surgical approaches for removal of ectopic third molars in the mandibular condyle. Study design: MEDLINE search of articles published on ectopic third molars in the mandibular condyle from 1980 to 2011. 14 well-documented clinical cases from the literature were evaluated together with a new clinical case provided by the authors, representing a sample of 15 patients. Results: We found a mean age at diagnosis of 48.6 years and a higher prevalence in women. In 14 patients, associated radiolucent lesions were diagnosed on radiographic studies and confirmed histopathologically as odontogenic cysts. Clinical symptoms were pain and swelling in the jaw or preauricular region, trismus, difficulty chewing, cutaneous fistula and temporomandibular joint dysfunction. Treatment included conservative management in one case and in the other cases, surgical removal by intra- or extraoral approaches, the latter being the most common approach carried out. In most reported cases, serious complications were not outlined. Conclusions: The etiopathogenic theory involving odontogenic cysts in the displacement of third molars to the mandibular condyle seems to be the most relevant. They must be removed if they cause symptoms or are associated with cystic pathology. The surgical route must be planned according to the location and position of the ectopic third molar, and the possible morbidity associated with surgery.

** Key words:**Third molar, ectopic tooth, condyle, mandible.

## Introduction

The finding of an impacted third molar in the mandible in an ectopic position displaced away from its usual anatomic position is an infrequent event. Very few cases have been reported in the literature; therefore, the knowledge about its etiology, clinical features, therapeutic options, and surgical approaches for extraction is limited (1). They have been described in ascending ramus, condylar region and coronoid process. The present study is based on a literature review of cases of ectopic third molars located in the region of the mandibular condyle reported from 1980 to 2011, together with the presentation of a clinical case taken from the personal experience of the authors.

## Material and Methods

An initial literature search in Medline/PubMed (http://www.ncbi.nlm.nih.gov/PubMed) including articles from January 1980 to June 2011 was conducted using the following keywords: “ectopic tooth”, “third molar” and “mandibular”. Inclusion criteria were: English-language articles generated by the Medline database containing at least one case of an ectopic third molar in the mandible, and articles comprising cases of patients with ectopic wisdom teeth located in the mandibular condyle that detailed clinical, diagnostic and therapeutic criteria. Among 56 papers found in the initial search, 32 met the criteria. Consequently, a search was conducted by dividing the papers into three sub-themes: “ectopic third molar and mandibular ramus”, “ectopic third molar and mandibular coronoid process” and “ectopic third molar and mandibular condyle”. We identified 2 articles about impacted third molar in the mandibular ramus, 10 articles in the coronoid process and 14 articles in the condylar/subcondylar region (1-16).

## Results

This study included 15 patients with ectopic third molar in the mandibular condyle: 14 well-documented clinical cases taken from the literature (5-16), along with a clinical case provided from the personal experience of the authors ( Table 1).

**Table 1:**
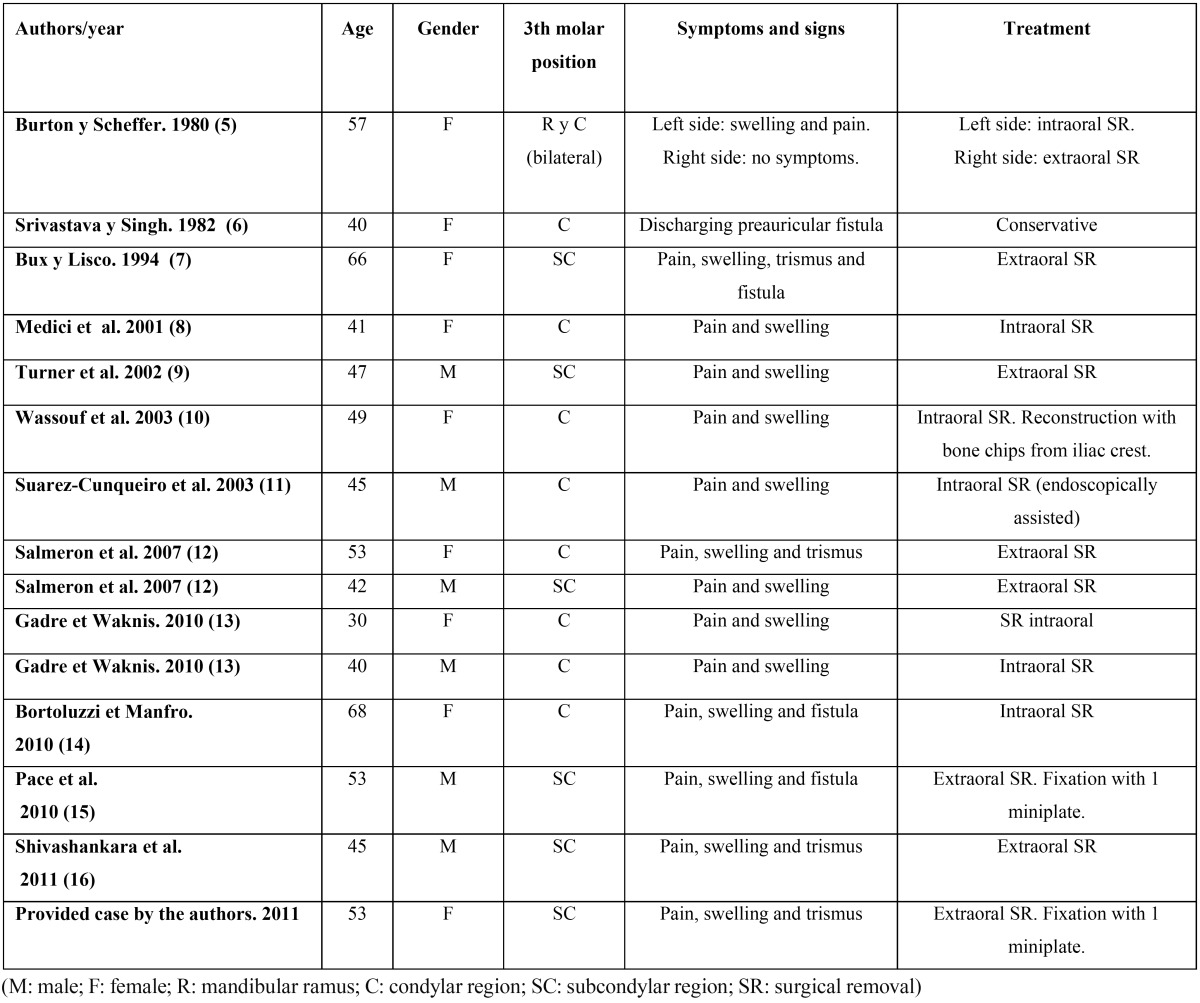
Ectopic third molars in the condylar region.

**Figure 1:**
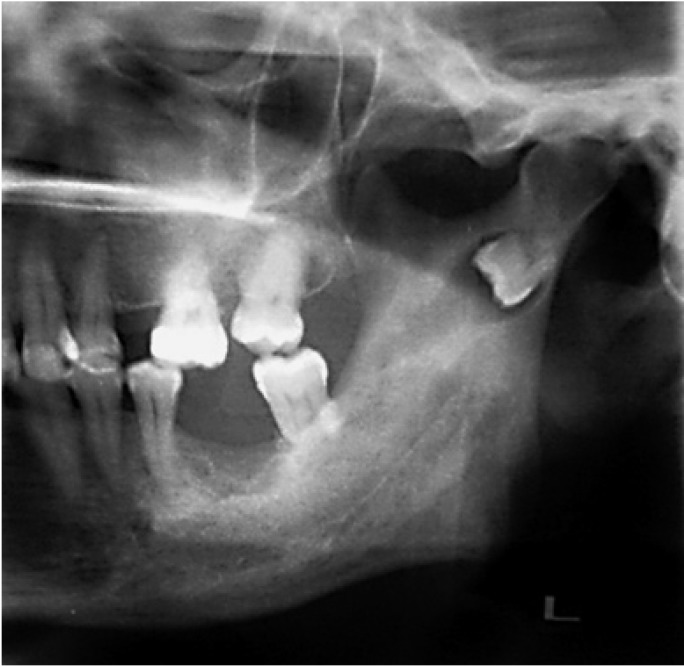
Panoramic radiograph showing an inverted third molar associated with a radiolucent image in the left subcondylar region.

**Figure 2:**
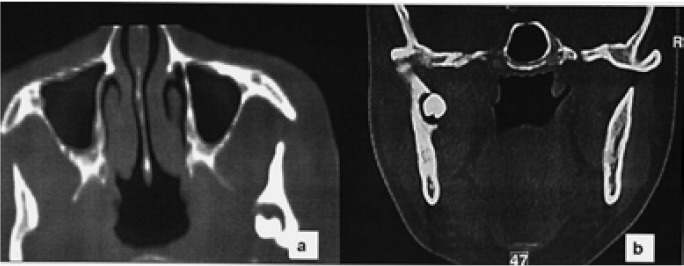
Axial a) and coronal b) TC showing the location of the third molar with an inverted position in the subcondilar region, with its crown facing downward marking on the internal cortical bone and a radiolucent area around.

**Figure 3:**
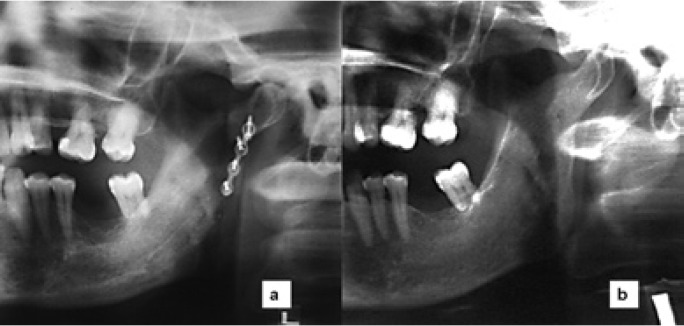
a) Postoperative panoramic radiograph showing one miniplate of osteosynthesis. b) Panoramic radiograph after 5 years of follow up.

The new clinical case reported here was a 53 years-old-woman who had suffered several episodes of intense pain and swelling in the left preauricular region accompanied by trismus, during the last year. The panoramic radiograph (Fig. 1) showed an ectopic third molar in the left condylar region in an inverted position, with a radiolucent image around the third molar crown. A continuation of the radiolucent image displayed toward the retromolar trigone simulating an abnormal eruption path. In the axial and coronal computed tomography (CT), the ectopic third molar could be seen in the condylar region placed on an invert position with the crown facing downward marking on the internal cortical bone. The crown was associated with a radiolucent area (Fig. 2). Extraction was performed under general anesthesia by an extraoral approach through a left retromandibular incision. After sectioning the masseter muscle, the condilar region was reached through subperiosteal dissection. A subcondylar osteotomy was performed using a Lindeman bur and chisel to access the third molar located in the internal cortical bone. After tooth extraction and curettage of the site, the fixation of the osteotomy fragments was carried out using an osteosynthesis with one miniplate and four screws. An elastic intermaxillary fixation was placed for 10 days. The histopathology report confirmed the presence of a dentigerous cyst. The third molar had tartar on its crown, as if it had ever been in mouth. As complication in the postoperative period, the patient had a mild paresis of the marginal branch of the facial nerve, being fully recovered in 6 weeks. The osteosynthesis plate was removed 6 months later. After 5 years, the patient has shown a very good evolution, being free of symptoms (Fig. 3).

Among the 15 patients reported in the present study ( Table 1), we found a higher prevalence in women, including 9 cases. The mean age was 48.6 years and median 47 years, with a range from the 30 years-old of the younger patient to 68 years-old of the older one. Third molars were located on the right side in 4 patients and on the left side in 10 of them. One patient had 2 bilateral ectopic mandibular third molars, one located in the condyle and the other in the mandibular ramus (5). A radiolucent image around the ectopic third molar was described in 14 patients by radiographic tests. All these images turned out to be dentigerous cysts, except one case which was not confirmed histopathologically (16). There has been reported a case located in the right man-dibular coronoid process associated with a keratocyst (17). The most frequent signs and symptoms were pain and swelling on ipsilateral side of the mandible or the preauricular region, trismus, difficulty chewing, cutaneous fistula, and temporomandibular joint dysfunction ( Table 1). Asymptomatic ectopia´s cases have also been reported (18,19). 14 patients underwent ectopic third molar extraction; 7 third molars were extracted using an intraoral approach (one of them was performed endoscopically assisted) and the other 8 wisdom teeth were extracted using an extraoral approach. In one patient it was decided not to remove the ectopic third molar and to conduct a follow-up, since during surgery it was found that the cause of cutaneous fistula of the patient be-longed to the parotid gland, and the third molar was not associated with a cystic pathology (6). In most patients there were not serious complications. In cases in which extraoral approach was used, mild transient paresis of the facial nerve branches were described.

## Discussion

The etiology of an ectopic tooth cannot always be determined (1). It may be associated with developmental disorders of jaws, pathological conditions or iatrogenia (4). Several theories have been put forward to explain ectopic locations of third molars, including the aberrant eruption, trauma, and ectopic formation of tooth germs (12). A third molar may be displaced a great distance from its habitual location because of an aborted eruption, a displacement due to lesions such as cysts or osseous tumours, or an alteration of the eruption due to a lesions such as odontogenic tumors (6-8). Most of reported cases were associated with radiolucent lesions in the panoramic radiograph and were confirmed histopathologically as dentigerous cysts. Therefore, the theory involving odontogenic cysts in the pathogenesis of ectopic third molar seems to be the most relevant. However, the causes are still not understood and the involvement of other factors in the pathogenesis cannot be ruled out.

From 1960 to 1980, 8 cases of third molar in the condylar region, ascending ramus or coronoid process were published (1), all of them associated with dentigerous cysts (14), and in one patient appeared 2 ectopic wisdom teeth bilaterally (1). In the period studied in our paper (1980-2011), there were found 15 patients with third molars in the condylar or subcondylar region (including the new case that the authors provide), 5 patients with wisdom teeth in the ascending ramus and 6 patients with wisdom teeth in the coronoid process. Thus, the location on subcondilar or condylar region becomes the most common site of lower ectopic third molars. However, considering the theory of displacement of molar through the jaw bone, it would be logical to contemplate the condylar position as the most extreme and rarest, and it would be expected to find out a higher frequency in different previous positions of the route. In order to answer this question, two hypotheses can be posed. Firstly, one can consider the fact that they remain asymptomatic for several years until the displacement of third molars to the condylar region would make appear the first clinical signs, and radiographic diagnosis could be established. Secondly, it should be kept in mind the possibility of under-reported cases in the literature, being ectopic third molars in the condylar position the most suitable to be published because of their rarity and uniqueness.

Diagnosis is based on clinical findings together with imaging tests, mainly panoramic radiograph and CT (12). Imaging tests are not only used for definitive diagnosis, but also for the assessment of possible associated pathology, for location and accurate position of the ectopic third molar, and for the most appropriate treatment planning. After diagnosis it can be decided whether removing third molars or not treating it (6), since not all third molars found in ectopic positions need to be removed (14). Ectopic third molars diagnosed during routine radiographic examinations that are not associated with any disease do not require treatment (1), and a regular follow-up of patients must be planned in such cases (18,19). It seems clear that all ectopic third molars should be treated if they cause symptoms (14), are associated with cystic pathology, or in order to prevent future complications (8). In the clinical case presented in this article, the reasons to indicate the surgical treatment were symptoms as pain, swelling and trismus along a year. Treatment of third molars in the condylar region is recommended to prevent infections if there is a follicular cyst, to minimize the risk of fracture in an anatomical area with a thin bone due to the cyst´s growth, and to avoid temporomandibular joint dysfunction (1,12).

The election of the surgical approach is basically linked to the experience and preference of surgeons. All authors agree that treatment should be carefully planned according to the location and position of the ectopic tooth and the morbidity associated with surgery with the aim of choosing the more conservative technique that produces the least possible trauma to the patient (14). In general, an intraoral approach is advocated whenever possible (10), to avoid visible scars and facial nerve injury (13,20). However, there are certain situations in which this type of intraoral approach is not appropriate due to the limited surgical field and the poor visualisation in inaccessible anatomical regions. In these cases, the use of an endoscope produces a magnified visualisation of the surgical field and may be a suitable option instead of the extraoral approach usually indicated in ectopic third molars in mandibular condyle (11).

In relation to extraoral approaches, the most frequently used access routes have been the submandibular and retromandibular as they provide a good surgical exposure especially to the body and ascending ramus, and with a higher difficulty, to the condylar region. They have a low rate of facial nerve paresis and the cutaneous scar is habitually cosmetic (8,9). The preauricular approach provides a better vision of the condyle; however, it often results in an anaesthetic scar. It is worth mentioning that in the literature has been only described the use of extraoral approach when the third molar was located in the condylar-subcondylar region (5,7,9,12,15,16) and not in other locations closer to the eutopic one. In the clinical case presented in this paper, the retromandibular approach allowed us to carry out the subcondylar osteotomy, the access to the internal cortical, the wisdom tooth extraction, and the removal of the cyst.

Complications are infrequent. Among them, there can be highlighted the risks of fractures, possible damage to nerve structures and components of the temporomandibular joint, and aesthetic aspects (1). In most reported cases serious complications were not outlined, and nerve injuries were transient, such as the case presented in this paper. Mandibular fractures can become serious complications especially when associated with nerve injury. This risk increase when a limited visibility approach is chosen and when a greater osteotomy is performed (21). It is useful to carry out osteosynthesis with miniplates for fixing bone fragments and prevent fractures in these areas of weakness (10,15). It can be performed with one miniplate, although whenever possible it is more stable to place two miniplates located on the anterior and posterior region of the condyle. Annual monitoring of patients is recommended in the first years after surgery to verify the complete ossification of the defect created, because this is a weak area with a high risk of fracture (12).

Based on the findings of this study, we can conclude that there have been reported more cases of ectopic third molars located in the mandibular condylar region than in the ascending ramus or coronoid process. Taking into account the new clinical case described here by the authors, there have been reported 15 well-documented patients in the literature, with a total of 15 ectopic wisdom teeth. However, the true incidence is still unknown and seems to be undervalued. Among the multiple etiopathogenic hypotheses, the theory involving the odontogenic cysts appears to be the most relevant. The symptoms presented by the patients include pain and swelling of the ipsilateral side of the mandible or the preauricular region, trismus, difficulty chewing, cutaneous fistula, and temporomandibular joint dysfunction. The treatment of third molars in the condylar region is divided into conservative and, in most cases, surgical removal by intra- or extraoral route, the latter being the approach most often used. The approach must be carefully planned according to the location and position of ectopic third molars and the possible morbidity associated with surgery, with the aim of choosing the more conservative technique that produces the minimum trauma to patients. In most reported cases serious complications were not outlined.
